# Optimizing Autologous Stem Cell Transplantation in Multiple Myeloma: The Significance of Pre-Transplant Controlling Nutritional Status Score

**DOI:** 10.3390/life15020289

**Published:** 2025-02-12

**Authors:** Sıdıka Gülkan Özkan, Suna Avcı, Ali Kimiaei, Seyedehtina Safaei, Yüksel Altuntaş, Aslı Yüksel Öztürkmen, Zeynep Aslı Durak, Sinem Özdemir, Mohammad Adeeb Abbara, Tuğba Ağyol, Mehmet Serdar Yıldız, Hasan Atilla Özkan

**Affiliations:** 1 Division of Hematology, Department of Internal Medicine, Faculty of Medicine, Bahçeşehir University, Sahrayi Cedit, Batman Street No:66, Kadikoy, Istanbul 34734, Turkey; drgulkan@gmail.com (S.G.Ö.); tinasafaei@outlook.com (S.S.); durakzeynepasli@gmail.com (Z.A.D.); adeeb.abbara00@gmail.com (M.A.A.); atillaozkan@yahoo.com (H.A.Ö.); 2 Division of Geriatrics, Department of Internal Medicine, Cerrahpasa Faculty of Medicine, Istanbul University, Istanbul 34093, Turkey; sunavci012@yahoo.com; 3 Division of Endocrinology, Department of Internal Medicine, Sisli Hamidiye Etfal Training and Research Hospital, Istanbul 34418, Turkey; yukselaltuntas@yahoo.com (Y.A.); dr.mserdaryildiz@gmail.com (M.S.Y.); 4 Division of Hematology, Department of Internal Medicine, Sivas Numune Hospital, Sivas 58040, Turkey; yagkbln@gmail.com; 5 Adult Hematology and Bone Marrow Transplantation Unit, Medical Park Göztepe Hospital, Istanbul 34730, Turkey; ozdemir_sinem@hotmail.com (S.Ö.); tubacaki85@hotmail.com (T.A.)

**Keywords:** Controlling Nutritional Status Score, multiple myeloma, autologous stem cell transplantation

## Abstract

Nutritional status is an important prognostic factor in patients with multiple myeloma (MM). The Controlling Nutritional Status (CONUT) score has shown promise in predicting outcomes in various malignancies; however, its role in autologous stem cell transplantation (ASCT) in patients with MM remains unclear. This study aimed to evaluate the significance of pre-transplant CONUT scores in predicting post-transplant engraftment kinetics and early complications in patients with MM undergoing ASCT. This single-center, retrospective study analyzed 59 multiple myeloma patients who underwent ASCT between 1 October 2022, and 1 July 2024. Pre-transplant CONUT scores were calculated, and their associations with various post-transplant outcomes were assessed using statistical analyses. Higher CONUT scores were independently associated with longer neutrophil engraftment times (*p* = 0.012). Patients who developed oral mucositis (OM) had significantly higher CONUT scores than those without OM (*p* = 0.028). A CONUT score cut-off of 2.5 demonstrated 100% sensitivity and 57.14% specificity in predicting OM (Area Under the Curve (AUC) 0.792, 95% CI: 0.654–0.930, *p* = 0.033). Our study demonstrates that a higher pre-transplant CONUT score is significantly associated with a delay in neutrophil engraftment and an increased risk of developing oral mucositis. These findings suggest that the CONUT score can serve as a valuable predictive tool for early post-transplant complications, thereby guiding targeted interventions and improving patient management.

## 1. Introduction

Multiple myeloma (MM), the second most common hematologic malignancy, is a plasma cell malignancy characterized by the presence of monoclonal immunoglobulin in the blood and urine, along with clinical signs of organ damage associated with MM, such as hypercalcemia, kidney dysfunction, anemia, and bone-related complications [1,2]. Advances in the treatment of multiple myeloma over the last two decades have led to sustained improvements in overall survival (OS) and progression-free survival (PFS) [2,3]. While autologous stem cell transplantation (ASCT) remains the cornerstone of treatment for eligible patients, optimizing outcomes and minimizing complications remain key challenges in clinical practice [3].

The International Staging System (ISS), revised ISS (R-ISS), and, more recently, the second revision of R-ISS (R2-ISS) are widely used for risk stratification in MM [4,5]. However, these systems may not fully capture the impact of nutritional status on patient outcomes. In recent years, the role of nutritional status in predicting treatment response and survival in various malignancies, including MM, has gained increasing attention [6]. Nutritional indices, such as the Controlling Nutritional Status (CONUT) score, Prognostic Nutritional Index (PNI), and Geriatric Nutritional Risk Index (GNRI), have demonstrated prognostic value in various hematologic malignancies [6,7]. The CONUT score offers a comprehensive assessment of a patient’s nutritional health by incorporating serum albumin, total lymphocyte count, and total cholesterol levels [8].

Recent studies have demonstrated the prognostic value of the CONUT score in MM patients, with higher scores associated with poorer overall survival and progression-free survival [9,10,11]. However, its specific role in predicting short-term outcomes and complications in patients with MM undergoing ASCT remains less welldefined.

Despite growing evidence supporting the prognostic value of CONUT scores in MM, limited research exists on their role in the context of ASCT, particularly across diverse populations [9]. This study aimed to address this gap by evaluating the significance of pre-transplant CONUT scores in predicting post-transplant outcomes, including engraftment kinetics and early complications, in MM patients undergoing ASCT at our center.

## 2. Methods

### 2.1. Study Design and Patient Population

This study was designed as a single-center retrospective analysis of medical records. The data of 75 patients diagnosed with MM who underwent their first autologous stem cell transplantation at the Adult Bone Marrow Transplant Unit of Bahcesehir University Medicalpark Göztepe Hospital between 1 October 2022, and 1 July 2024 were analyzed retrospectively.

The inclusion criteria were as follows:

- Patients diagnosed with MM undergoing their first ASCT
- Availability of complete pre-transplant laboratory data, including serum albumin, cholesterol, and lymphocyte counts required for CONUT score calculation
- No use of anti-cholesterol medications in the six months prior to ASCT

The exclusion criteria were as follows:

- Use of anti-cholesterol medications within six months before ASCT
- Missing or incomplete laboratory data needed for CONUT score calculation
- Second or subsequent ASCT procedures

A total of 59 patients met all the inclusion criteria, and none of the exclusion criteria were included in the final analysis. Patients taking anti-cholesterol medications were excluded from the study because these agents can significantly alter the serum cholesterol level, which is a key component of the CONUT score. Excluding these patients minimizes potential confounding factors, ensuring that the CONUT score more accurately reflects the patients’ nutritional status. This study was conducted in compliance with the Declaration of Helsinki and approved by the local Institutional Ethics Committee on 8 August 2024 (approval number 4476). All participants provided written informed consent.

### 2.2. Treatment Protocols and Supportive Care

Melphalan 140 mg/m^2^ (MEL 140 mg/m^2^) or 200 mg/m^2^ (MEL 200 mg/m^2^) (divided into two doses on days -3 and -2) was used as a conditioning regimen, depending on whether the patients had renal impairment. This adjustment was based on established protocols to minimize toxicity in patients with compromised renal function [12]. Oral cryotherapy, antiseptic and antifungal mouthwash, and mucosamine mouthwash and spray were used for oral mucositis (OM) prophylaxis.

### 2.3. Follow-Up Methodologies

Given the retrospective design of the study, follow-up data were extracted from the patients’ electronic medical records. These data included records of scheduled clinical visits, laboratory test results (including nutritional markers and routine blood work), imaging studies, and any other diagnostic evaluations performed as part of the standard care protocol.

### 2.4. Engraftment Definitions and Complication Assessment

Neutrophil engraftment is defined as the first of three consecutive days with a sustained peripheral blood neutrophil count exceeding 500 × 10^6^/L. Platelet engraftment is determined by the first three consecutive days with a platelet count of 20,000/μL or higher, without the need for platelet transfusions for at least seven consecutive days [13]. All complications were classified and assessed using the Common Terminology Criteria for Adverse Events (CTCAE) version 5.0 [14]. The severity of oral mucositis was graded according to the World Health Organization (WHO) scale, ranging from 0 to 4 [15].

### 2.5. CONUT Score Calculation

The CONUT score was retrospectively calculated for each patient using the pre-transplant laboratory values of serum albumin (g/dL), total lymphocyte count (cells/mm^3^), and total cholesterol (mg/dL). The score was determined by assigning points to each parameter based on predefined thresholds: serum albumin ≥ 3.5 (0 points), 3.0–3.49 (2 points), 2.5–2.99 (4 points), or <2.5 (6 points); total lymphocyte count ≥ 1600 (0 points), 1200–1599 (1 point), 800–1199 (2 points), or <800 (3 points); and total cholesterol ≥ 180 (0 points), 140–179 (1 point), 100–139 (2 points), or <100 (3 points). The total CONUT score was calculated by summing the points for all three parameters, with scores interpreted as follows: 0–1 (normal nutritional status); 2–4 (mild malnutrition); 5–8 (moderate malnutrition); and 9–12 (severe malnutrition) [8].

### 2.6. Statistical Analysis

According to the CONUT score Receiver Operating Characteristic (ROC) analysis, patients were divided into two groups with a cut-off point of 2.5. Those with 2.5 and above were considered malnourished. Logistic regression analysis was performed to obtain odds ratios (OR) and 95% confidence intervals (CI) of the associations between nutritional status, early complications, and engraftment times.

IBM SPSS Statistics for Windows, Version 25.0 (IBM Corp., Armonk, NY, USA) was used for statistical analysis. The Shapiro-Wilk test was used to examine the conformity of the variables to a normal distribution. Descriptive statistics were presented using the median (25th percentile–75th percentile) for continuous variables due to the non-normality of distribution and frequency (percentage) for categorical variables. The relationships between the variables and engraftment times were evaluated using Spearman’s correlation coefficient. Multivariable linear regression analysis was used to determine factors independently associated with neutrophil engraftment time. Statistically significant factors, according to the correlation coefficients, were included in the multivariable linear regression analysis. Continuous variables were analyzed using the Mann−Whitney U test due to the non-normality of the distribution. Categorical variables were analyzed using Fisher’s exact test or the Fisher−Freeman−Halton test. The oral mucositis prediction performance of the CONUT score was assessed using ROC curve analysis. The optimal cut-off point was determined using Youden’s index. Two-tailed *p*-values of less than 0.05 were considered statistically significant. We utilized Receiver Operating Characteristic (ROC) analysis to determine the optimal cut-off point for the CONUT score. This method was selected because our primary outcome is dichotomous, and ROC analysis allows for an effective balance between sensitivity and specificity. Moreover, the application of the Youden index in this context provides a robust criterion for cut-off selection, as it maximizes the difference between true-positive and false-positive rates.

## 3. Results

We included 59 patients (22 females and 37 males) in the study; the median age at transplantation was 61 years (interquartile range, 54–65 years; range, 29–76 years). Six (10.17%) patients had OM, and 13 (22.03%) patients had other complications. None of the patients experienced primary or secondary graft failure. Two (3.39%) patients experienced relapses during follow-up, and none of the cases were fatal. The median follow-up time after transplantation was 3 months (interquartile range, 2–7 months; range, 1–13 months) (Table 1).

Neutrophil engraftment time was positively correlated with male sex (r = 0.299, *p* = 0.022), Karnofsky Performance Status (KPS) score (r = 0.407, *p* = 0.001), Hematopoietic Cell Transplantation Comorbidity Index (HCT-CI) score (r = 0.288, *p* = 0.027), and CONUT score (r = 0.432, *p* = 0.001). Platelet engraftment time was positively correlated with the KPS score (r = 0.287, *p* = 0.027) (Table 2).

**Figure 1:**
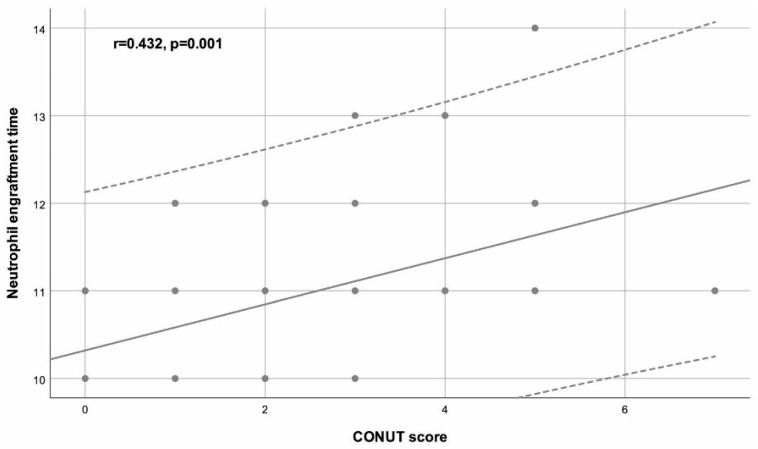
The scatter plot shows a positive correlation between the Controlling Nutritional Status (CONUT) score and neutrophil engraftment time in the study population (r = 0.432, *p* = 0.001). Each point represents an individual patient with higher CONUT scores associated with longer neutrophil engraftment times.

Figure 1 illustrates the positive correlation between the CONUT score and neutrophil engraftment time.

According to the results of the multivariable linear regression analysis, male sex (*p* = 0.040), high HCT-CI score (*p* = 0.043), and high CONUT score (*p* = 0.012) were independently associated with higher neutrophil engraftment time (Table 3).

**Figure 2:**
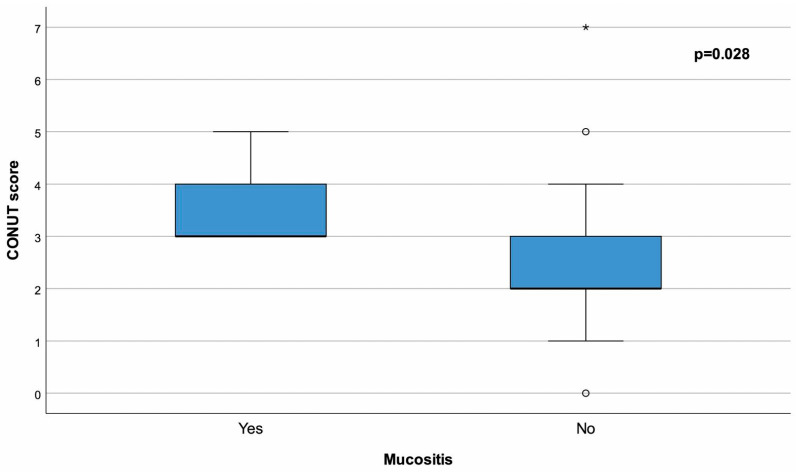
The box plots compare the controlling nutritional status (CONUT) scores between patients with and without OM. Patients who developed OM had significantly higher CONUT scores compared to those who did not (*p* = 0.028).

The time between diagnosis and transplantation was significantly higher in the patients with complications group than in the other groups (*p* = 0.048). Additionally, the CONUT score was significantly higher in the patients with the OM group compared to the other groups (*p* = 0.028) (Figure 2). However, no significant differences were found between the complication and OM groups in terms of age at transplantation, sex, ISS, R-ISS, Karnofsky Performance Status (KPS) score, ECOG Performance Status score, HCT-CI score, disease status at transplantation, stem cell count, conditioning regimen, time between diagnosis and transplantation, neutrophil engraftment time, platelet engraftment time, relapse, and follow-up time (Table 4 and Table 5).

**Figure 3:**
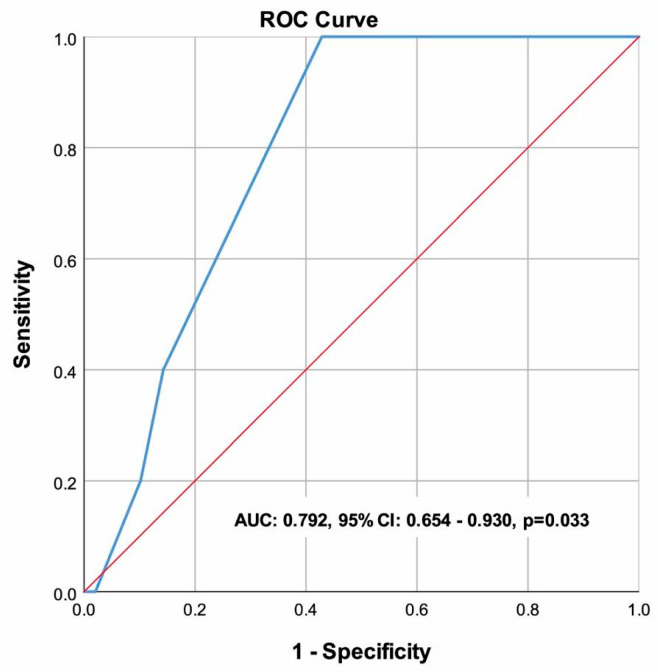
The Receiver Operating Characteristic (ROC) curve illustrates the ability of the Controlling Nutritional Status (CONUT) score to predict oral mucositis (OM), with an area under the curve (AUC) of 0.792 (95% CI: 0.654–0.930, *p* = 0.033). A CONUT score cut-off of 2.5 provided 100.00% sensitivity and 57.14% specificity for predicting OM.

The CONUT score demonstrated a strong predictive capability for oral mucositis, with a cut-off value of 2.5, yielding a sensitivity of 100% and a specificity of 57.14%. The negative predictive value was 100%, indicating that patients with a CONUT score below 2.5 had no risk of developing OM, while the positive predictive value was 19.23%, reflecting a lower probability of accurately identifying OM cases. The area under the ROC curve was 0.792 (95% CI: 0.654–0.930, *p* = 0.033), confirming the CONUT score’s potential utility as a predictive tool for OM in patients with MM undergoing ASCT (Figure 3).

Table 6 shows the ROC curve analysis of the CONUT score for predicting oral mucositis.

## 4. Discussion

In this study, we investigated the significance of nutritional status with pre-transplant CONUT score, an age-independent malnutrition index, in optimizing ASCT for MM patients. We found that a higher CONUT score was independently associated with prolonged neutrophil engraftment time and higher OM. To our knowledge, our study is the first to identify that the CONUT score has 100.00% sensitivity and 100.00% negative predictive value to predict OM with a cut-off point of 2.5 (with higher scores predicting OM). Our findings highlight the importance of nutritional status in predicting early transplant outcomes.

The incidence of OM in patients undergoing hematopoietic stem cell transplantation (HSCT) has been reported to range from 35% to 75%, affecting autologous transplant recipients [16,17]. Specifically, for patients with MM receiving melphalan-based ASCT, studies have shown OM rates of 75%, with 21–46% experiencing severe forms, particularly in those with elevated serum creatinine levels and high melphalan doses [18,19]. In contrast, our study demonstrated a notably lower incidence of OM (10.17%), which can be attributed to several factors, including enhanced patient care, fractionated melphalan dosing, cryotherapy application, and the use of mucosamin. This finding represents a significant difference from those of previous studies and contributes valuable insights to the literature on reducing OM risk. While previous studies have identified risk factors such as low albumin and elevated creatinine levels, other studies have found no predictive factors, suggesting that pharmacogenetic investigations of melphalan metabolism may offer insights into individual predispositions to OM [18,20]. Moreover, to the best of our knowledge, our study is the first to identify a higher CONUT score as an independent predictor of increased OM incidence in patients with MM undergoing ASCT. This novel finding underscores the importance of nutritional status not only in transplant outcomes but also in the risk of post-transplant complications such as OM. 

Engraftment times in ASCT are primarily influenced by the infused CD34+ cell dose, with higher doses leading to faster engraftment [21]. Other factors affecting engraftment include the quality of infused cells, patient characteristics such as age and race, chemotherapy-induced marrow damage, infections, and disease relapse [21]. While Xiong et al. [22] found no significant differences in hematopoietic recovery or adverse reactions between the high and low CONUT groups, our study identified a higher CONUT score as independently associated with delayed neutrophil engraftment. This discrepancy may be due to differences in patient populations, transplant protocols, or other factors, warranting further investigation. Our study contributes uniquely to the literature by being the first to establish an association between CONUT score and neutrophil engraftment time in MM patients undergoing ASCT.

In our study, we observed that a prolonged interval between diagnosis and transplantation was significantly associated with an increased incidence of early complications, predominantly infections. This finding aligns with some prior studies that have suggested the potential benefits of early transplantation in terms of response rates and progression-free survival [23,24]. However, the relationship between the time of transplantation and complications is likely multifactorial. Factors such as the intensity and duration of pre-transplant therapy, the number of treatment cycles administered, periods of treatment delay, and underlying conditions like hypogammaglobulinemia may contribute to this association. Given our study’s limited sample size and follow-up duration, it remains challenging to ascertain whether the extended time to transplant is a direct cause of the observed complications or a surrogate marker for other confounding clinical factors.

Recent studies have demonstrated the prognostic value of CONUT scores in patients with multiple myeloma. Okamoto et al. [9] found that transplant-eligible patients with high CONUT scores (>4) showed worse overall survival than those with low scores (≤4). Similarly, Kamiya et al. [10] reported that patients with high CONUT scores (≥5) had a significantly shorter median overall survival compared to those with low scores (33 months vs. 57 months, *p* < 0.001). These findings were further validated in Zhou et al.’s [11] larger study of 245 patients, which showed declining 5-year overall survival rates across CONUT score groups: 65.1% for low scores (≤3), 38.9% for medium scores (4–9), and 16.6% for high scores (>9). Additionally, Xiong et al. [22] specifically examined pre-transplant CONUT scores in patients undergoing ASCT and found that patients with low scores (<5) achieved superior overall survival and progression-free survival compared to those with high scores (≥5). While our study focused on short-term outcomes and had no mortality during follow-up, these studies consistently demonstrate that elevated CONUT scores serve as an independent prognostic factor for poor survival in MM patients, particularly in younger transplant-eligible patients and those with lower ISS scores [10].

While most studies support the prognostic value of the CONUT score in MM, Liang et al. found that it was not an independent factor, arguing that age and plasma cell ratio were more significant prognostic indicators [25]. The relationship between poor nutritional status and worse clinical outcomes in MM is complex, involving interactions between nutrition, inflammation, and the myeloma microenvironment [26]. Albumin, a component of both the CONUT score and ISS, can be influenced by inflammatory cytokines like IL-6 from the myeloma microenvironment and changes in body fluid volume [26]. Malnutrition leads to impaired immune function, reduced hematopoietic recovery, and increased systemic inflammation, which can negatively impact post-transplant recovery [27,28]. The CONUT score’s incorporation of multiple nutritional parameters, including hypoalbuminemia and cholesterol levels, provides added value in assessing patients’ overall health status, as these factors influence immune modulation, cell membrane integrity, and transplant outcomes.

This study has several limitations that should be considered. First, because this was a single-center retrospective analysis, the findings may be subject to selection bias and may not be generalizable to other populations. Reliance on medical records can introduce inaccuracies. In addition, the relatively short follow-up period may not have been sufficient to fully capture any long-term effects. Another limitation of our approach is its inherent dependence on the chosen outcome measure for the ROC analysis. Although the ROC method is widely accepted, it may not capture the full complexity of the relationship between CONUT scores and long-term outcomes. It is also important to note that the time between diagnosis and transplantation may reflect other underlying factors, such as disease severity or comorbid conditions; therefore, the observed association with complications may not be entirely direct. Although our sample size was sufficient to detect significant associations, we acknowledge that no formal power analysis was conducted. Future studies should incorporate power calculations to ensure optimal sample size determination and further validate our findings.

In conclusion, our study adds to the growing body of evidence supporting the utility of CONUT score in assessing nutritional status and predicting outcomes in patients with MM undergoing autologous stem cell transplantation. The significant associations we found between the CONUT score and neutrophil engraftment time, as well as its predictive value for OM, suggest that this simple and readily available tool could be valuable in pre-transplant risk assessment and management strategies. Future prospective studies with larger patient cohorts and longer follow-up periods are needed to further validate these findings and explore the potential benefits of nutritional interventions based on CONUT scores in improving transplant outcomes and long-term survival in patients with MM.

## Figures and Tables

**Table 1:** Summary of variables.

Age at transplantation (n = 59)	61 (54–65)
Sex (n = 59)	
Female	22 (37.29%)
Male	37 (62.71%)
ISS (n = 47)	
1	17 (36.17%)
2	15 (31.91%)
3	15 (31.91%)
R-ISS (n = 33)	
1	5 (15.15%)
2	21 (63.64%)
3	7 (21.21%)
Karnofsky Performance Status score (n = 59)	90 (80–90)
ECOG Performance Status score (n = 59)	0 (0–1)
HCT-CI score (n = 59)	0 (0–2)
CONUT score (n = 54)	2 (2–3)
Disease status at transplantation (n = 53)	
CR	24 (45.28%)
VGPR	18 (33.96%)
PR	11 (20.75%)
Stem cell count (×10^6^/kg) (n = 54)	5.69 (4.60–7.00)
Conditioning regimen (n = 59)	
MEL 200 mg/m^2^	44 (74.58%)
MEL 140 mg/m^2^	15 (25.42%)
Time between diagnosis and transplantation, months (n = 59)	7 (5–11)
Neutrophil engraftment time (n = 59)	11 (10–11)
Platelet engraftment time (n = 59)	12 (10–14)
Primary graft failure (n = 59)	0 (0.00%)
Complication (n = 59)	13 (22.03%)
CMV viremia	1 (1.69%)
Klebsiella bacteremia	1 (1.69%)
*E. coli* bacteremia	2 (3.39%)
*E. coli* bacteremia + atrial fibrillation	1 (1.69%)
Febrile neutropenia	5 (8.47%)
Diarrhea	1 (1.69%)
Epileptic seizure	1 (1.69%)
Transient ischemic attack	1 (1.69%)
Oral Mucositis (n = 59)	6 (10.17%)
Grade 1	3 (5.08%)
Grade 2	2 (3.39%)
Grade 3	1 (1.69%)
Grade 4	0 (0.00%)
Relapse (n = 59)	2 (3.39%)
Mortality (n = 59)	0 (0.00%)
Follow-up time after transplantation, months (n = 59)	3 (2–7)

Descriptive statistics are presented using the median (25th percentile–75th percentile) for non-normally distributed continuous variables and frequency (percentage) for categorical variables. ISS: International Staging System; R-ISS: Revised International Staging System; ECOG: Eastern Cooperative Oncology Group; HCT-CI: Hematopoietic Cell Transplantation Comorbidity Index; CONUT: Controlling Nutritional Status; CR: Complete Response; VGPR: Very Good Partial Response; PR: Partial Response; MEL: Melphalan; CMV: Cytomegalovirus; *E. coli*: *Escherichia coli*.

**Table 2:** Correlations between engraftment time and other variables.

		Neutrophil Engraftment Time	Platelet Engraftment Time
Age at transplantation	r	−0.069	−0.008
	*p*	0.604	0.949
Sex, Male	r	0.299	0.085
	*p*	0.022	0.524
ISS	r	0.033	0.253
	*p*	0.826	0.086
R-ISS	r	−0.170	−0.043
	*p*	0.344	0.814
Karnofsky Performance Status score	r	0.407	**0.287**
	*p*	0.001	**0.027**
ECOG Performance Status score	r	0.170	0.210
	*p*	0.199	0.110
HCT-CI score	r	0.288	0.191
	*p*	0.027	0.147
CONUT score	r	0.432	0.165
	*p*	0.001	0.234
Disease status at transplantation	r	−0.132	−0.043
	*p*	0.346	0.761
Stem cell count (×10^6^/kg)	r	0.088	0.034
	*p*	0.526	0.806
Conditioning regimen, MEL140 mg/m^2^	r	0.208	0.165
	*p*	0.114	0.213
Time between diagnosis and transplantation, months	r	−0.110	−0.012
	*p*	0.405	0.929

r: correlation coefficient; *p*: *p*-value (statistical significance); ISS: International Staging System; R-ISS: Revised International Staging System; ECOG: Eastern Cooperative Oncology Group; HCT-CI: Hematopoietic Cell Transplantation Comorbidity Index; CONUT: Controlling Nutritional Status; MEL: Melphalan.

**Table 3:** Associations between factors and neutrophil engraftment time, multivariable linear regression analysis.

	Unstandardized β	Standard Error	Standardized β	*p*	95% Confidence Interval for β	
(Constant)	7.812	1.162		<0.001	5.477	10.147
Sex, Male	0.487	0.231	0.250	**0.040**	0.023	0.951
Karnofsky Performance Status score	0.020	0.014	0.174	0.179	−0.009	0.049
HCT-CI score	0.166	0.080	0.243	**0.043**	0.006	0.327
CONUT score	0.207	0.080	0.329	**0.012**	0.047	0.367

β: regression coefficient; *p*: *p*-value (statistical significance); R^2^: coefficient of determination; F: F-statistic. R^2^ = 0.297; F = 6.596; *p* < 0.001; HCT-CI: Hematopoietic Cell Transplantation Comorbidity Index; CONUT: Controlling Nutritional Status.

**Table 4:** Summary of variables with regard to complication.

	Complication		
	Yes (n = 13)	No (n = 46)	*p*
Age at transplantation	61 (54–64)	60 (54–65)	0.769 ^‡^
Sex			
Female	6 (46.15%)	16 (34.78%)	0.524 ^§^
Male	7 (53.85%)	30 (65.22%)	
ISS			
1	4 (33.33%)	13 (37.14%)	0.286 ^¶^
2	2 (16.67%)	13 (37.14%)	
3	6 (50.00%)	9 (25.71%)	
R-ISS			
1	1 (12.50%)	4 (16.00%)	0.434 ^¶^
2	4 (50.00%)	17 (68.00%)	
3	3 (37.50%)	4 (16.00%)	
Karnofsky Performance Status score	90 (90–90)	90 (80–90)	0.612 ^‡^
ECOG Performance Status score	0 (0–1)	0 (0–1)	0.655 ^‡^
HCT-CI score	1 (0–2)	0 (0–2)	0.766 ^‡^
CONUT score	2 (2–4)	2 (2–3)	0.778 ^‡^
Disease status at transplantation			
CR	6 (46.15%)	18 (45.00%)	1.000 ^¶^
VGPR	4 (30.77%)	14 (35.00%)	
PR	3 (23.08%)	8 (20.00%)	
Stem cell count (×10^6^/kg)	6.03 (5.40–7.30)	5.60 (4.60–6.68)	0.442 ^‡^
Conditioning regimen			
MEL 200 mg/m^2^	10 (76.92%)	34 (73.91%)	1.000 ^§^
MEL 140 mg/m^2^	3 (23.08%)	12 (26.09%)	
Time between diagnosis and transplantation, months	9 (7–13)	6 (4–8)	**0.048 ^‡^**
Neutrophil engraftment time	11 (10–11)	11 (10–11)	0.695 ^‡^
Platelet engraftment time	13 (10–15)	12 (10–13)	0.214 ^‡^
Relapse	0 (0.00%)	2 (4.35%)	1.000 ^§^
Follow-up time after transplantation, months	3 (2–4)	3.5 (2–7)	0.839 ^‡^

CR: Complete Response; VGPR: Very Good Partial Response; PR: Partial Response; HCT-CI: Hematopoietic Cell Transplantation Comorbidity Index; ECOG: Eastern Cooperative Oncology Group; CONUT: Controlling Nutritional Status; MEL: Melphalan; *p*: *p*-value (statistical significance). Descriptive statistics were presented using median (25th percentile–75th percentile) for non-normally distributed continuous variables and frequency (percentage) for categorical variables. ^‡^ Mann Whitney U test, ^§^ Fisher’s exact test, ^¶^ Fisher-Freeman Halton test.

**Table 5:** Summary of variables with regard to oral mucositis.

	Oral Mucositis		
	Yes (n = 6)	No (n = 53)	*p*
Age at transplantation	63.5 (61–65)	59 (53–65)	0.187 ^‡^
Sex			
Female	3 (50.00%)	19 (35.85%)	0.661 ^§^
Male	3 (50.00%)	34 (64.15%)	
ISS			
1	3 (60.00%)	14 (33.33%)	0.599 ^¶^
2	1 (20.00%)	14 (33.33%)	
3	1 (20.00%)	14 (33.33%)	
R-ISS			
1	1 (25.00%)	4 (13.79%)	0.593 ^¶^
2	3 (75.00%)	18 (62.07%)	
3	0 (0.00%)	7 (24.14%)	
Karnofsky Performance Status score	90 (80–90)	90 (80–90)	0.882 ^‡^
ECOG Performance Status score	1 (1–1)	0 (0–1)	0.090 ^‡^
HCT-CI score	2 (0–3)	0 (0–2)	0.241 ^‡^
CONUT score	3 (3–4)	2 (2–3)	0.028 **^‡^**
Disease status at transplantation			
CR	2 (40.00%)	22 (45.83%)	0.591 ^¶^
VGPR	1 (20.00%)	17 (35.42%)	
PR	2 (40.00%)	9 (18.75%)	
Stem cell count (×10^6^/kg)	5.80 (4.60–7.50)	5.68 (4.70–7.00)	0.811 ^‡^
Conditioning regimen			
MEL 200 mg/m^2^	4 (66.67%)	40 (75.47%)	0.638 ^§^
MEL 140 mg/m^2^	2 (33.33%)	13 (24.53%)	
Time between diagnosis and transplantation, months	4 (1–15)	7 (6–9)	0.520 ^‡^
Neutrophil engraftment time	11 (11–12)	11 (10–11)	0.375 ^‡^
Platelet engraftment time	12 (12–14)	12 (10–13)	0.327 ^‡^
Relapse	0 (0.00%)	2 (3.77%)	1.000 ^§^
Follow-up time after transplantation, months	5.5 (3–9)	3 (2–6)	0.144 ^‡^

CR: Complete Response; VGPR: Very Good Partial Response; PR: Partial Response; HCT-CI: Hematopoietic Cell Transplantation Comorbidity Index; ECOG: Eastern Cooperative Oncology Group; CONUT: Controlling Nutritional Status; MEL: Melphalan; *p*: *p*-value (statistical significance). Descriptive statistics were presented using median (25th percentile–75th percentile) for non-normally distributed continuous variables and frequency (percentage) for categorical variables. ^‡^ Mann Whitney U test, ^§^ Fisher’s exact test, ^¶^ Fisher-Freeman Halton test.

**Table 6:** Performance of CONUT score in predicting OM, ROC curve analysis.

Cut-off	>2.5
Sensitivity	100.00%
Specificity	57.14%
Accuracy	61.11%
PPV	19.23%
NPV	100.00%
AUC (95% CI)	0.792 (0.654–0.930)
*p*	0.033

ROC, Receiver Operating Characteristic; PPV: Positive Predictive Value; NPV: Negative Predictive Value; AUC: Area Under the ROC Curve; CI: Confidence Interval; *p*, *p*-value (statistical significance).

## Data Availability

The data presented in this study are available upon request from the corresponding author due to ethical restrictions.

## References

[1] Ozaki S., Handa H., Saitoh T., Murakami H., Itagaki M., Asaoku H., Suzuki K., Isoda A., Matsumoto M., Sawamura M. (2019). Evaluation of the Revised International Staging System (R-ISS) in Japanese patients with multiple myeloma. Ann. Hematol..

[2] Shafiei F.S., Abroun S. (2024). Recent advancements in nanomedicine as a revolutionary approach to treating multiple myeloma. Life Sci..

[3] Devarakonda S., Efebera Y., Sharma N. (2021). Role of Stem Cell Transplantation in Multiple Myeloma. Cancers.

[4] Greipp P.R., San Miguel J., Durie B.G., Crowley J.J., Barlogie B., Blade J., Boccadoro M., Child J.A., Avet-Loiseau H., Kyle R.A. (2005). International staging system for multiple myeloma. J. Clin. Oncol..

[5] Rajkumar S.V., Dimopoulos M.A., Palumbo A., Blade J., Merlini G., Mateos M.V., Kumar S., Hillengass J., Kastritis E., Richardson P. (2014). International Myeloma Working Group updated criteria for the diagnosis of multiple myeloma. Lancet Oncol..

[6] Matsukawa T., Suto K., Kanaya M., Izumiyama K., Minauchi K., Yoshida S., Oda H., Miyagishima T., Mori A., Ota S. (2020). Validation and comparison of prognostic values of GNRI, PNI, and CONUT in newly diagnosed diffuse large B cell lymphoma. Ann. Hematol..

[7] Mancuso S., Mattana M., Santoro M., Carlisi M., Buscemi S., Siragusa S. (2022). Host-related factors and cancer: Malnutrition and non-Hodgkin lymphoma. Hematol. Oncol..

[8] Ignacio de Ulibarri J., Gonzalez-Madrono A., de Villar N.G., Gonzalez P., Gonzalez B., Mancha A., Rodriguez F., Fernandez G. (2005). CONUT: A tool for controlling nutritional status. First validation in a hospital population. Nutr. Hosp..

[9] Okamoto S., Ureshino H., Kidoguchi K., Kusaba K., Kizuka-Sano H., Sano H., Nishioka A., Yamaguchi K., Kamachi K., Itamura H. (2020). Clinical impact of the CONUT score in patients with multiple myeloma. Ann. Hematol..

[10] Kamiya T., Ito C., Fujita Y., Ogura S., Mizuno K., Sakurai A., Aisa Y., Nakazato T. (2020). The prognostic value of the controlling nutritional status score in patients with multiple myeloma. Leuk. Lymphoma.

[11] Zhou X., Lu Y., Xia J., Mao J., Wang J., Guo H. (2021). Association between baseline Controlling Nutritional Status score and clinical outcomes of patients with multiple myeloma. Cancer Biomarks.

[12] Brown Z., Scott C., Zhang L.F., Sadek R., Clarke A., Jillella A., Keruakous A.R., Clemmons A.B. (2024). Assessing Outcomes in Patients with Multiple Myeloma Postautologous Stem Cell Transplantation: Contrasting the Effects of Melphalan Dosages at 200 mg/m^2^ versus 140 mg/m^2^. Clin. Lymphoma Myeloma Leuk..

[13] Kharfan-Dabaja M.A., Kumar A., Ayala E., Aljurf M., Nishihori T., Marsh R., Burroughs L.M., Majhail N., Al-Homsi A.S., Al-Kadhimi Z.S. (2021). Standardizing Definitions of Hematopoietic Recovery, Graft Rejection, Graft Failure, Poor Graft Function, and Donor Chimerism in Allogeneic Hematopoietic Cell Transplantation: A Report on Behalf of the American Society for Transplantation and Cellular Therapy. Transplant. Cell. Ther..

[14] National Cancer I. (2017). Common Terminology Criteria for Adverse Events (CTCAE) Version 5.0.

[15] Miller A.B., Hoogstraten B., Staquet M., Winkler A. (1981). Reporting results of cancer treatment. Cancer.

[16] Moreau P., Facon T., Attal M., Hulin C., Michallet M., Maloisel F., Sotto J.J., Guilhot F., Marit G., Doyen C. (2002). Comparison of 200 mg/m^2^ melphalan and 8 Gy total body irradiation plus 140 mg/m^2^ melphalan as conditioning regimens for peripheral blood stem cell transplantation in patients with newly diagnosed multiple myeloma: Final analysis of the Intergroupe Francophone du Myelome 9502 randomized trial. Blood.

[17] Alhussain A., Alkhayal Z., Ayas M., Abed H. (2022). Prevalence and risk factors of oral mucositis in paediatric patients undergoing haematopoietic stem cell transplantation. Oral Dis..

[18] Grazziutti M.L., Dong L., Miceli M.H., Krishna S.G., Kiwan E., Syed N., Fassas A., van Rhee F., Klaus H., Barlogie B. (2006). Oral mucositis in myeloma patients undergoing melphalan-based autologous stem cell transplantation: Incidence, risk factors and a severity predictive model. Bone Marrow Transplant..

[19] Blijlevens N., Schwenkglenks M., Bacon P., D’Addio A., Einsele H., Maertens J., Niederwieser D., Rabitsch W., Roosaar A., Ruutu T. (2008). Prospective oral mucositis audit: Oral mucositis in patients receiving high-dose melphalan or BEAM conditioning chemotherapy—European Blood and Marrow Transplantation Mucositis Advisory Group. J. Clin. Oncol..

[20] Bhatnagar B., Goloubeva O.G., Gilmore S., Hoffman A., Ruehle K., Akpek G., Rapoport A., Yanovich S., Badros A.Z. (2012). Risk factors for oral mucositis (OM) in multiple myeloma (MM) patients receiving high-dose melphalan (Mel) prior to autologous stem cell transplantation (SCT). J. Clin. Oncol..

[21] Lutfi F., Skelton Iv W.P., Wang Y., Rosenau E., Farhadfar N., Murthy H., Cogle C.R., Brown R., Hiemenz J., Wingard J.R. (2020). Clinical predictors of delayed engraftment in autologous hematopoietic cell transplant recipients. Hematol. Oncol. Stem Cell Ther..

[22] Xiong Y.Y., Zhou Q., Chen L., Yu W., Zhang H.B., Chen J.B. (2024). Effects of Pre-Transplant CONUT and Post-Transplant MRD on Prognosis of Patients with Multiple Myeloma after Auto-HSCT. Zhongguo Shi Yan Xue Ye Xue Za Zhi.

[23] Perrot A., Lauwers-Cances V., Cazaubiel T., Facon T., Caillot D., Clement-Filliatre L., Macro M., Decaux O., Belhadj K., Mohty M. (2020). Early Versus Late Autologous Stem Cell Transplant in Newly Diagnosed Multiple Myeloma: Long-Term Follow-up Analysis of the IFM 2009 Trial. Blood.

[24] Kumar L., Hussain M.M., Chethan R., Sahoo R.K., Malik P.S., Sharma O.D., Mathew A., Jha A., Gupta R., Sharma A. (2022). Multiple Myeloma: Impact of Time to Transplant on the Outcome. Clin. Lymphoma Myeloma Leuk..

[25] Liang F., Dong X.Y., Tang G.F., Qi K.M., Chen W., Sang W., Sun H.Y., Cao J., Cheng H., Li D.P. (2021). Influence of prognostic nutritional index and controlling nutritional status on the prognosis of patients with multiple myeloma. Zhonghua Xue Ye Xue Za Zhi.

[26] Yang Q.K., Su Y.N., Wang W., Wang N., Yao Z.X., Zhang X.J. (2020). CONUT Score or/and Peripheral Blood CD4+/CD8+ Ratio-Based Web Dynamic Nomograms to Predict the Individualized Survival of Patients with Advanced Osteosarcoma. Cancer Manag. Res..

[27] Bourke C.D., Berkley J.A., Prendergast A.J. (2016). Immune Dysfunction as a Cause and Consequence of Malnutrition. Trends Immunol..

[28] Morello E., Guarinoni M.G., Arena F., Andreoli M., Bernardi S., Malagola M., Turra A., Polverelli N., Russo D. (2020). A Systematic Review of the Literature and Perspectives on the Role of Biomarkers in the Management of Malnutrition After Allogeneic Hematopoietic Stem Cell Transplantation. Front. Immunol..

